# Algorithm Versus Expert: Machine Learning Versus Surgeon-Predicted Symptom Improvement After Carpal Tunnel Release

**DOI:** 10.1227/neu.0000000000002848

**Published:** 2024-02-01

**Authors:** Nina Louisa Loos, Lisa Hoogendam, John Sebastiaan Souer, Jeroen Hein van Uchelen, Harm Pieter Slijper, Robbert Maarten Wouters, Ruud Willem Selles

**Affiliations:** * Department of Rehabilitation Medicine, Erasmus MC, Rotterdam, The Netherlands;; ‡ Department of Plastic and Reconstructive Surgery and Hand Surgery, Erasmus MC, Rotterdam, The Netherlands;; § Hand and Wrist Center, Xpert Clinics, Eindhoven, The Netherlands

**Keywords:** Prediction, Carpal tunnel release, BCTQ, Hand surgery, Machine learning, Carpal tunnel syndrome

## Abstract

**BACKGROUND AND OBJECTIVES:**

Surgeons rely on clinical experience when making predictions about treatment effects. Incorporating algorithm-based predictions of symptom improvement after carpal tunnel release (CTR) could support medical decision-making. However, these algorithm-based predictions need to outperform predictions made by surgeons to add value. We compared predictions of a validated prediction model for symptom improvement after CTR with predictions made by surgeons.

**METHODS:**

This cohort study included 97 patients scheduled for CTR. Preoperatively, surgeons estimated each patient's probability of improvement 6 months after surgery, defined as reaching the minimally clinically important difference on the Boston Carpal Tunnel Syndrome Symptom Severity Score. We assessed model and surgeon performance using calibration (calibration belts), discrimination (area under the curve [AUC]), sensitivity, and specificity. In addition, we assessed the net benefit of decision-making based on the prediction model's estimates vs the surgeon's judgement.

**RESULTS:**

The surgeon predictions had poor calibration and suboptimal discrimination (AUC 0.62, 95%-CI 0.49-0.74), while the prediction model showed good calibration and appropriate discrimination (AUC 0.77, 95%-CI 0.66-0.89, *P* = .05). The accuracy of surgeon predictions was 0.65 (95%-CI 0.37-0.78) vs 0.78 (95%-CI 0.67-0.89) for the prediction model (*P* = .03). The sensitivity of surgeon predictions and the prediction model was 0.72 (95%-CI 0.15-0.96) and 0.85 (95%-CI 0.62-0.97), respectively (*P* = .04). The specificity of the surgeon predictions was similar to the model's specificity (*P* = .25). The net benefit analysis showed better decision-making based on the prediction model compared with the surgeons' decision-making (ie, more correctly predicted improvements and/or fewer incorrectly predicted improvements).

**CONCLUSION:**

The prediction model outperformed surgeon predictions of improvement after CTR in terms of calibration, accuracy, and sensitivity. Furthermore, the net benefit analysis indicated that using the prediction model instead of relying solely on surgeon decision-making increases the number of patients who will improve after CTR, without increasing the number of unnecessary surgeries.

**ABBREVIATIONS:**
BCTQ: Boston Carpal Tunnel Questionnaire
CTR: carpal tunnel release
CTS: carpal tunnel syndrome
DCA: decision curve analysis
MCID: minimal clinically important difference
PROM: patient-reported outcome measure
SSS: Symptom Severity Scale

Surgical treatment of carpal tunnel syndrome (CTS) generally has superior outcomes than nonsurgical treatment.^1^ Although surgical treatment is low-risk and generally successful,^2,3^ not all patients improve, and for some, returning to work takes months.^2,4^ Therefore, predicting the individual probability of a successful outcome would be useful. This will help inform patients about treatment options, supporting decision-making of both patients and surgeons. This may help increase patient satisfaction and treatment success and avoid unnecessary healthcare consumption.^5,6^

We previously developed a prediction model to predict the probability of patient-reported symptom improvement after carpal tunnel release (CTR).^7^ The model predicts the patients' probability of achieving a clinically relevant improvement 6 months after surgery, using 5 patient-reported outcome measure (PROM) scores as predictors. In temporal validation, our model showed good calibration and appropriate discriminative ability with an area under the curve (AUC) of 0.71. The model is available online.^8^

Besides sufficient performance in internal and external validation, determining the clinical usefulness of prediction models is important, but often overlooked.^9^ Several factors contribute to the success of prediction models in clinical practice^10-13^: (1) whether the model's predictions are more accurate than predictions made by clinicians, (2) how prediction results are integrated into clinical practice, and (3) use and acceptance of prediction models by clinicians. Furthermore, for prediction models to be of added value, they should result in better medical decision-making. This study aims to assess our prediction model's clinical value by comparing its performance with the performance of surgeons predicting improvement after CTR and evaluate the effect on decision-making.

## PATIENTS AND METHODS

We conducted a prospective observational study at Xpert Clinics Hand and Wrist Care between January 1st and October 13th, 2021. Xpert Clinics comprises 25 locations for hand and wrist care in The Netherlands, employs 23 European Board (Federation of European Societies for Surgery of the Hand)–certified hand surgeons, and offers a hand surgery fellowship program. Patients treated at Xpert Clinics are invited to routinely complete PROMs. This cohort and data collection have been described elsewhere.^14^ The study was approved by the local institutional review board and reported according to The Strengthening the Reporting of Observational Studies in Epidemiology Statement.^15^ All patients provided informed consent.

We considered patients scheduled for CTR eligible for inclusion. Patients who did not complete the Boston Carpal Tunnel Questionnaire (BCTQ) before treatment and 6 months postoperatively, were excluded, since these scores were used to assess clinical improvement.

Patients were diagnosed and treated by hand surgeons. Diagnoses were based on clinical symptoms, findings on physical examination, and nerve conduction studies when required. All patients underwent a mini-open CTR and received standard postoperative care.^16^

Our primary outcome was the Symptom Severity Scale (SSS) of the BCTQ, and additionally, we used visual analog scales (VAS) to assess hand function and pain. The BCTQ is specifically developed for CTS. It consists of the SSS and the functional status scale. Both scales range from 1 to 5, with higher scores representing more severe CTS.^17^ Because relieving symptoms (tingling or pain) is generally most important, we focused on the SSS.^18^ We used the minimal clinically important difference (MCID) of 0.8 to define a clinically relevant improvement.^19^

In addition, patients were invited to complete PROMs on their mindset toward their condition and treatment, and on their mental health. These questionnaires included the Brief Illness Perception Questionnaire, the Credibility and Expectancy questionnaire, the Pain Catastrophizing Scale, and the Patient Health Questionnaire-4.^20-23^ All PROMs were sent via email after the first consultation.

The prediction model for clinical improvement after CTR requires 5 subscales of previously mentioned PROMs at baseline as predictors: the BCTQ-SSS, VAS hand function score, Credibility and Expectancy questionnaire Expectancy Score, Patient Health Questionnaire-4 depression score, and the Brief Illness Perception Questionnaire illness comprehension score. These predictors were selected through recursive feature elimination from multiple potential predictors, including demographic factors and PROM scores.^7^ Missing data on these items were imputed through K-nearest neighbors by the prediction model. The predictions were made retrospectively, but were based on PROMs completed by patients before surgery (ie, the model only had access to preoperative data).

Surgeons received information on the study protocol, the prediction model, the BCTQ-SSS, and the MCID. After the consultation but before the point where patients completed the baseline PROMs, surgeons answered 3 questions on their expectations about the improvement of each new patient scheduled for CTR. Surgeons completed these questions on the day of the first consultation.

1. What do you think the patient scores on the SSS at this moment (from 1 to 5)?
2. What do you think the patient will score on the SSS 6 months after surgery (from 1 to 5)?
3. What is, according to you, the probability that the patient will improve with at least the MCID (0.8 points) on the SSS, 6 months after surgery?

The first 2 questions assessed the surgeons' understanding of the SSS, because insufficient understanding might affect their predictions. The third question was used for comparison with model predictions.

Six months after surgery, patients were invited to complete the BCTQ and VAS pain and function. Satisfaction with treatment result was assessed as part of routine outcome measurements.^24^ Patients failing to complete the BCTQ after 2 email reminders were contacted by phone once. The difference between the SSS at intake and 6 months after surgery was calculated to determine whether the patient reached a clinically relevant symptom improvement.

We aimed to detect a difference in accuracy of 80% correct model predictions and 70% correct surgeon predictions with a power of 0.80 and a 2-sided alpha of 0.05. This resulted in a sample size of 146 patients. To account for potential dropout, we aimed for a sample size of approximately 200 patients. However, our inclusion stopped prematurely because of an administrative change in the workflow, preventing us to collect the surgeon predictions in the same way. Therefore, the study inclusion was stopped before reaching the required sample size.

We performed a nonresponder analysis comparing patients with complete BCTQ scores and patients with missing BCTQ scores at baseline and 6 months postoperatively. *T*-tests were used for normally distributed variables and Wilcoxon tests were used for non-normally distributed variables.

We used the Wilcoxon signed rank test to compare surgeons' estimates of the SSS with observed scores at baseline and 6 months after surgery. Paired *t*-tests were used to compare estimated SSS change scores to observed scores.

We evaluated the prediction model's and surgeons' performance using calibration, discrimination, and accuracy. Calibration measures the accordance between predicted probabilities and observed frequencies of events. This indicates whether, for example, of 10 patients with a predicted probability of 0.6, we observe that 6 patients actually improve. We considered calibration the most important outcome, because using estimations from models with poor calibration can lead to misinforming patients on the likely success of a treatment.^25^ Calibration was visually assessed using calibration belts.^26^ Discrimination refers to the ability to distinguish between patients who will improve and patients who will not.^27^ Discrimination was assessed with the AUC. Similar to previous literature, we considered an AUC below 0.70 as suboptimal, 0.70–0.79 as good, and equal or above 0.80 as excellent.^28^ We used the DeLong's test to compare the AUCs of the model and of surgeons.^29^ Accuracy refers to the percentage of correctly predicted outcomes (ie, improved or not improved). Accuracy, sensitivity, and specificity were compared using McNemar's test.

Finally, the net benefit of using the prediction model was assessed using decision curve analysis (DCA).^30^ With DCA, we evaluated the effect of different decision-making strategies: (1) “treat none,” (2) “current decision-making,” and (3) “deciding based on the prediction model.” Current practice indicates that surgeons identify patients eligible for CTR based on their expertise. DCA compares the net benefit of each strategy, combining the benefits and harms associated with each strategy, calculated as a weighted difference between the true positives and false positives. True positives represent cases where the decision-maker correctly identified patients who benefit from CTR, while false positives indicate cases where the decision-maker suggested CTR, but patients did not benefit. A higher net benefit indicates that a decision strategy results in more true positives or fewer false positives than other strategies. This implies that more patients who truly benefit from surgery are correctly scheduled for CTR, while fewer patients who will not benefit from CTR are incorrectly scheduled for CTR.^30^ The net benefit is calculated across a range of threshold probabilities, representing the predicted probability at which the surgeon is willing to choose one option (eg, CTR) over the other (eg, no CTR). So, in other words, it reflects the point at which the benefits of a decision outweigh the potential risks. A threshold of 10% indicates that the surgeon feels the benefits of the CTR outweigh the risks if the patient has more than 10% chance of improvement after CTR. The threshold probability varies depending on individual perspectives and the specific context of the decision. For example, a higher threshold probability indicates that surgeons are more cautious and require a higher level of confidence before choosing CTR.

Analyses were performed using R statistical programming (version 4.2.2). Statistical significance was determined at *P* < .05.

## RESULTS

During the study period, 205 patients were eligible for inclusion. Ninety-seven patients completed the BCTQ before surgery and 6 months postoperatively and were included for analysis (**Supplemental Digital Content 1**, http://links.lww.com/NEU/E99). No significant differences were found in the nonresponder analysis (**Supplemental Digital Content 2**, http://links.lww.com/NEU/E100).

The mean age of included patients was 57 years (SD 12) and 66% were female (Table 1). The mean preoperative BCTQ-SSS was 2.9 (SD 0.7) (**Supplemental Digital Content 3**, http://links.lww.com/NEU/E101). Patients who reached the MCID were more satisfied with their treatment result and had better VAS pain and function scores (**Supplemental Digital Content 4**, http://links.lww.com/NEU/E102).

**TABLE 1.** Characteristics of the Included Patients

Characteristics	Included patients (n = 97)
Age (in y)	57.0 (12.4)
Sex, n (%)	
Female	64 (66)
Duration of symptoms (in mo)	9.0 [6.0, 24.0]
Type of work, n (%)	
Unemployed	34 (35)
Light physical labor	29 (30)
Moderate physical labor	21 (22)
Heavy physical labor	13 (13)
Affected side, n (%)	
Left	41 (42)
Right	55 (57)
Both	1 (1)
Recurrent CTS, n (%)	
Yes^a^	3 (3)
Second opinion, n (%)	
Yes	1 (1)

CTS, carpal tunnel syndrome.

a Patients with recurrent CTS were previously treated elsewhere.

Twelve hand surgeons and 1 fellow participated in the study. Ninety-two percent were male and 85% were trained as a plastic surgeon (Table 2). Surgeons were Federation of European Societies for Surgery of the Hand–certified, or fellows trained in hand surgery.

**TABLE 2.** Characteristics of the Participating Hand Surgeons

Characteristics	Participating surgeons (N = 13)
Sex, n (%)	
Female	1 (8)
Years of experience as specialist	9.69 (5.25)
Type of employment, n (%)	
Fellow hand surgery	1 (8)
Trained hand surgeon	12 (92)
Type of training, n (%)	
Plastic surgery	11(85)
Orthopedic surgery	2 (15)
Solely focused on hand surgery	
Yes	9 (70)
No	4 (30)
European board (FESSH) certified	
Yes	12 (96)
No	1 (8)

FESSH, Federation of European Societies for Surgery of the Hand.

Surgeons overestimated the preoperative symptom severity of patients (Wilcoxon effect size 0.61, *P* < .001) (**Supplemental Digital Content 5**, http://links.lww.com/NEU/E103). There was no difference between the surgeon-predicted and observed SSS 6 months after surgery (Wilcoxon effect size 0.10, *P* = .90). The surgeon-predicted improvement from baseline to 6 months after surgery was larger than the observed improvement (Cohen's d 0.94, *P* < .001). This indicates that surgeons believed patients had more severe symptoms before surgery and that surgeons predicted a greater improvement after surgery than was observed.

**FIGURE 1.**
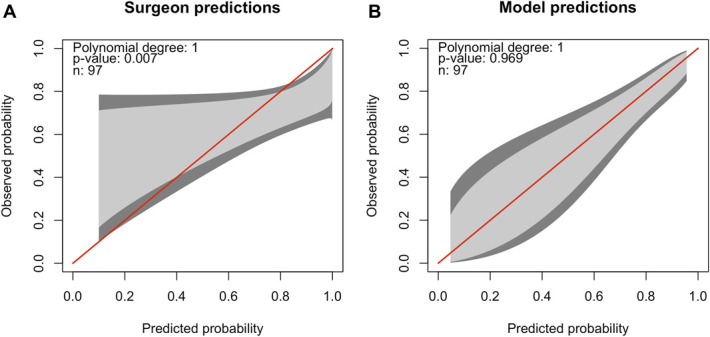
Calibration belts of **A**, the surgeon predictions and **B**, the prediction model. The predicted probability of symptom improvement compared with the observed probability of symptom improvement is shown. The red line indicates perfect calibration, and the 80% CI (light gray) and 95% CI (dark gray) of the calibration is shown in gray. The model or surgeons perform well on calibration if the belt is close to the bisector. If the belt falls above the bisector, the model or surgeons underestimate the probability of improvement. If the belt falls under the bisector, the model or surgeons overestimate the probability. **A**, Surgeon predictions: the red line does not fall within the 80% CI (light gray) and 95% CI (dark gray) over the whole range of predicted probability, indicating significant deviation in calibration between the predicted probability and observed probability of improvement. This is also confirmed by the *P*-value <.05. Patients for whom surgeons predict the probability of symptom improvement to be higher than 80%, the observed probability of symptom improvement is lower, indicating an overly optimistic surgeon prediction. **B**, Prediction model: the red line falls within the 80% CI (light gray) and 95% CI (dark gray), indicating no significant deviation in calibration between the predicted probability and observed probability of improvement. This is also confirmed by the *P*-value >.05.

The model's calibration curve did not show any deviations, indicating good calibration, while we found that the surgeon predictions had poor calibration (Figure 1). Specifically, the surgeons' calibration curve deviated at predicted probabilities below 0.3 and above 0.8. This means that surgeon predictions with probabilities of symptom improvement below 0.3 or above 0.8 are unreliable.

**FIGURE 2.**
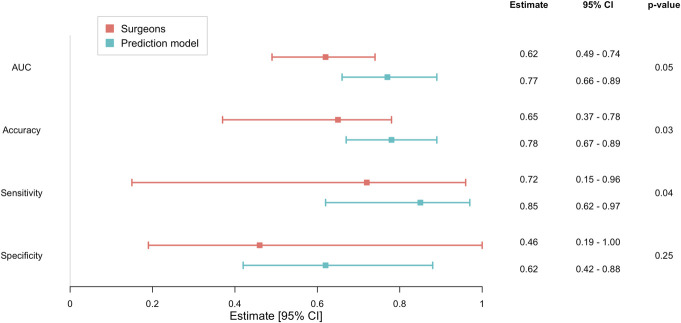
Comparison of performance measures (AUC, accuracy, sensitivity, and specificity) of surgeon predictions and model predictions. Higher scores indicate better performance. For all measures, estimates are displayed with 95% CI. In addition, the *P*-value for the comparisons between surgeon and model predictions is shown. A significantly higher sensitivity for the prediction model compared with surgeon predictions is seen. AUC, area under the curve.

Accuracy (0.78 (95%-CI 0.67-0.89) vs 0.65 (95%-CI 0.37-0.78), *P* = .03) and sensitivity (0.85 (95%-CI 0.62-0.97) vs 0.72 (95%-CI 0.15-0.96), *P* = .04) were higher for the prediction model (Figure 2). However, DeLong's test showed no difference in discriminative ability in surgeon (AUC 0.62, 95%-CI 0.49-0.74) and model predictions (AUC 0.77, 95%-CI 0.66-0.89, *P* = .05). Similarly, we found no difference in specificity of the model (0.62, 95%-CI 0.42-0.88) compared with specificity of surgeons (0.46, 95%-CI 0.19-1.00, *P* = .25).

**FIGURE 3.**
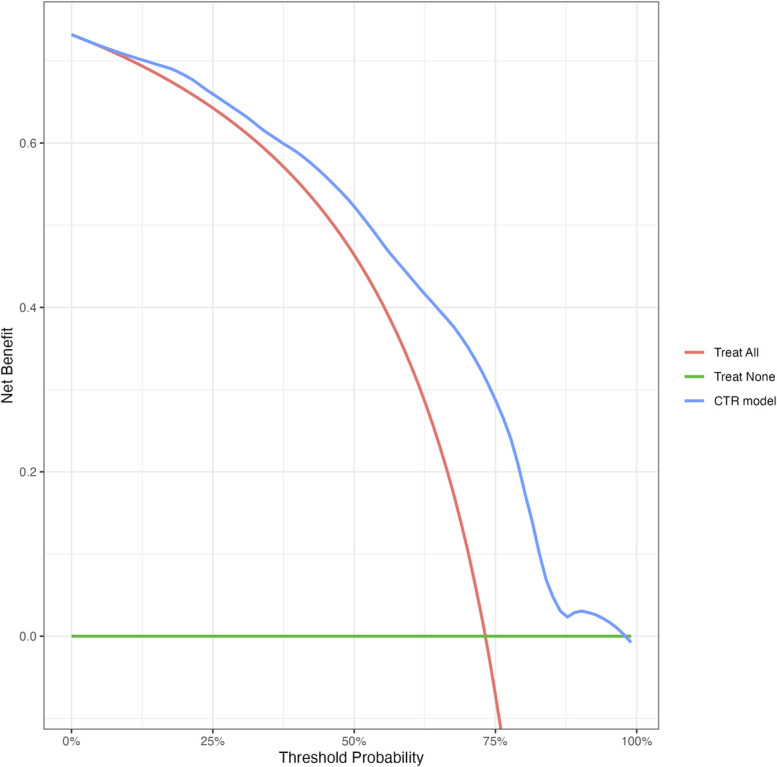
Net benefit curve for 3 decision-making strategies: “Treat all,” “Treat none,” and “Deciding based on the CTR prediction model.” “Treat all” is, in our sample equal, to the current decision-making of surgeons. The net benefit weighs the benefits (ie, true positives) and harms (ie, false positives) of a decision strategy over a range of threshold probabilities. The threshold probability (on the x-axis) reflects the point at which the benefits of a particular decision or strategy outweigh the potential harm. We observe a benefit of “Deciding based on the CTR prediction model” compared with “Treat all” from a threshold probability of 10% onward. A threshold of 10% would indicate that the surgeon feels the benefits of the CTR outweigh the risks if the patient has more than 10% chance of improvement after CTR. Given the elective nature of CTR, it is likely that surgeons would only schedule their patients for this procedure when the patient has a high probability of improving. Therefore, it is likely that the threshold probability for choosing CTR lies above 10%. The higher net benefit of “Deciding based on the CTR prediction model” indicates that this strategy results in more true positives and/or less false positives compared with current decision-making (“Treat all”). CTR, carpal tunnel release.

The DCA showed a higher net benefit of “deciding based on the prediction model” compared with current decision-making (“Treat all;” Figure 3).

## DISCUSSION

We compared surgeon predictions to a previously developed prediction model for patient-reported symptom improvement after CTR. Our model outperformed surgeons in terms of calibration, sensitivity, and accuracy. Surgeons overestimated the baseline symptom severity of patients and the treatment effect after surgery. Furthermore, the DCA showed better decision-making when using the model instead of relying solely on surgeon expertise. This suggests that our model could be of added value in clinical practice by providing individual information on expected outcomes and supporting decision-making. Model performance was similar to the performance we found in previous temporal validation.^7^

Evaluating patients and informing them of the expected outcomes of CTR is routine for surgeons, so it is likely they perform well on this task. Consequently, for a prediction model to be valuable, it should at least perform equally well as, and preferably better than, surgeons.

The model had access to several PROM scores, while the surgeons do not systematically collect this information, which could be considered an unequal comparison. However, surgeons usually have years of experience in assessing the prognosis and symptom severity of their patients, know the actual patient behind the data, and generally obtain more detailed anamnestic and diagnostic information from their consultations and physical examination, such as whether thenar atrophy is present. Therefore, we believe that both the model and surgeons have access to sufficient relevant data to be able to predict the probability of improvement.

Although there is ample research on the development of prediction models for outcomes after treatment, we were unable to find previous studies assessing the value of prognostic prediction models by comparing the model-based predictions with surgeon estimates. There is, however, some research on this subject for other applications, such as for medical imaging,^31,32^ outcomes, such as predicting patient survival in cancer patients,^33,34^ disease progression,^35^ and complications after cardiothoracic surgery.^36^ In a systematic review on prediction models in medical imaging for diagnostics, Nagendran et al^31^ found that most studies reported prediction models to have similar or superior performance compared with surgeons, with only 2 studies reporting prediction models performing worse. Similarly, the results of Kuo et al^32^ suggest that prediction models are not only noninferior to surgeons in diagnostic performance in fracture detection on medical imaging, but also that surgeon performance improved with the assistance of an artificial intelligence–based prediction model.

The results of this study were presented and discussed with the participating surgeons. Surgeons indicated that, for standard procedures, they are confident to rely on their own knowledge and experience to educate patients about the expected improvement. Therefore, they only find prediction models useful when they are uncertain about the treatment outcome. The prediction model may also have more difficulty predicting the probability of improvement for these relatively difficult cases. Future research should evaluate whether the prediction model has added benefit for these cases. However, our results indicate that, even for a standard procedure such as CTR, using the prediction model resulted in improved decision-making compared with relying only on the surgeons' expertise for all patients. In addition, surgeons indicated that they sometimes schedule surgery to prevent (further) nerve damage, instead of symptom improvement. Finally, surgeons expressed skepticism about prediction models' ability to capture the entire context of a patient's situation. However, we do not intend for prediction models to replace the shared decision-making, but rather to complement this process by providing individualized information. We believe prediction models can be valuable in patient education, enabling patients to become more involved.

### Limitations

We were unable to reach our intended sample size of 146 because of an administrative change in workflow, resulting in our study being underpowered. Therefore, we consider our findings “preliminary conclusions” and recommend repeating this study in a larger sample. Our model predicts the probability of reaching an improvement of 0.8 on the BCTQ-SSS, which may not be the best indicator for a successful outcome. However, patients who improved beyond 0.8 points were more likely to be satisfied with their treatment result and had less pain 6 months after surgery, suggesting it can be considered as an appropriate indicator of successful outcome.

We only included patients who were scheduled for CTR. In our clinics, there are differences in PROMs depending on whether patients are treated surgically or nonsurgically. Consequently, the BCTQ at the relevant time points for comparison with predictions was only available for patients scheduled for CTR. Limiting the study to patients scheduled for surgery could affect the surgeons' responses because of cognitive dissonance avoidance, “I choose to perform CTR, therefore it must help.” If surgeons were less sure, they could induce nocebo in patients. Future studies should evaluate the clinical benefit and external validity of the prediction model in a broader population of symptomatic patients with CTS. This will provide valuable information for the assessment of clinical usefulness and implementation.

## CONCLUSION

Predicting clinical improvement after CTR could help manage patient expectations and improve preoperative patient selection. This preliminary study comparing surgeon predictions of symptom improvement after CTR to model predictions indicated that surgeons showed a worse performance than our model, based on calibration, accuracy, and sensitivity. The DCA indicated that if surgeons would use our prediction model during clinical practice, they would more frequently select patients who will benefit from surgery without increasing the number of unnecessary procedures. Although future studies with larger sample sizes are needed to validate our findings, we believe our results show that prediction models are promising tools with added value for decision support.

## Supplementary Material

SUPPLEMENTARY MATERIAL

## Supplementary Material

**Figure:**
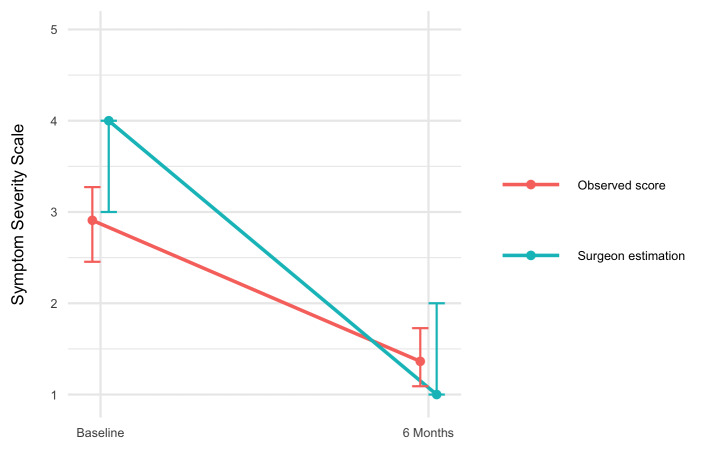


## SUPPLEMENTAL DIGITAL CONTENT

**Supplemental Digital Content 1. Figure 1.** Flowchart of patient selection. **Supplemental Digital Content 2. Table 1.** Nonresponder analysis. **Supplemental Digital Content 3. Table 2.** Preoperative Patient-Reported Outcome Scores of the included patients. Note that these were collected after the first consultation with the surgeon, making the surgeon blinded to these values. The values indicated with an asterisk are used in the prediction model. **Supplemental Digital Content 4. Table 3.** VAS scores and satisfaction scores 6 months after surgery of patients who reached the MCID and those who did not, to provide context on the relevance of the MCID. NB: Not all included patients completed all follow-up questionnaires, which resulted in some missing data in the VAS scores and satisfaction scores 6 months after surgery. **Supplemental Digital Content 5. Figure 2.** Observed Symptom Severity Scores (SSS) vs surgeon-estimated (baseline) and surgeon-predicted (follow-up) scores. Surgeons estimated a median baseline SSS of 4.0 [IQR 3.0-4.0] compared to a median observed SSS of 2.9 [IQR 2.5-3.3] (*P* < .001), indicating that surgeons overestimated preoperative symptom severity. Surgeons predicted that patients would score a median of 1.0 [IQR 1.0-2.0] 6 months after surgery, while the observed score was 1.4 [IQR 1.1-1.7] (*P* = .90).
