# Supplementary figures and images for: Algorithm Versus Expert: Machine Learning Versus Surgeon-Predicted Symptom Improvement After Carpal Tunnel Release
Source: Neurosurgery. 2024 Feb 1;95(1):110–7. doi: 10.1227/neu.0000000000002848 (PMC11155572; doi:10.1227/neu.0000000000002848)

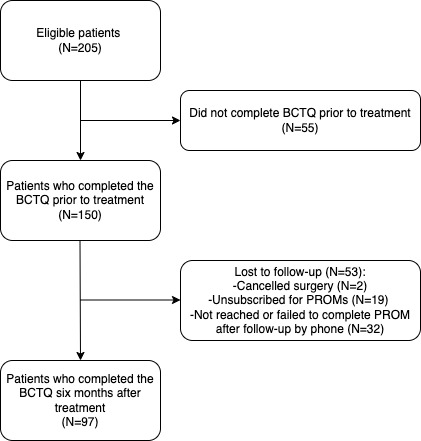

Supplement: SUPPLEMENTARY MATERIAL [file neu-95-110-s001.jpg]
