# Supplementary material for: Algorithm Versus Expert: Machine Learning Versus Surgeon-Predicted Symptom Improvement After Carpal Tunnel Release
Source: Neurosurgery. 2024 Feb 1;95(1):110–7. doi: 10.1227/neu.0000000000002848 (PMC11155572; doi:10.1227/neu.0000000000002848)
Supplement: SUPPLEMENTARY MATERIAL [file neu-95-110-s003.docx]

***Supplementary Table 1***. Non-responder analysis

|  | Non-responders  (n = 108) | Responders  (n = 97) | p-value | SMD |
| --- | --- | --- | --- | --- |
| Age (in years) | 55.9 (15.1) | 57.0 (12.4) | 0.56 | 0.082 |
| Gender, n (%) |  |  |  | 0.005 |
| Female | 71 (66) | 64 (66) | 1.00 |  |
| Duration of symptoms (in months) | 8.50 [5.00, 24.00] | 9.0 [6.0, 24.0] | 0.54 | 0.13 |
| Type of work, n (%) |  |  | 0.26 | 0.28 |
| Unemployed | 37 (34) | 34 (35) |  |  |
| Light physical labor | 22 (20) | 29 (30) |  |  |
| Moderate physical labor | 35 (32) | 21 (22) |  |  |
| Heavy physical labor | 14 (13) | 13 (13) |  |  |
| Affected side, % (n) |  |  | 0.45 | 0.17 |
| Left | 41 (38) | 41 (42) |  |  |
| Right | 67 (62) | 55 (57) |  |  |
| Ambidextrous | 0 (0) | 1 (1) |  |  |
| Recurrent CTS, n (%) |  |  | 0.61 | 0.12 |
| Yes | 6 (6) | 3 (3) |  |  |
| Second opinion, n (%) |  |  |  | 0.011 |
| Yes | 1 (1) | 1 (1) | 1.0 |  |
