# Supplementary material for: Algorithm Versus Expert: Machine Learning Versus Surgeon-Predicted Symptom Improvement After Carpal Tunnel Release
Source: Neurosurgery. 2024 Feb 1;95(1):110–7. doi: 10.1227/neu.0000000000002848 (PMC11155572; doi:10.1227/neu.0000000000002848)
Supplement: SUPPLEMENTARY MATERIAL [file neu-95-110-s004.docx]

***Supplementary Table 2.*** Preoperative Patient-Reported Outcome Scores of the included patients. Note that these were collected after the first consultation with the surgeon, making the surgeon blinded to these values. The values indicated with an asterisk are used in the prediction model.

|  | Included patients (n=97) | Percentage missing |
| --- | --- | --- |
| BCTQ |  |  |
| Symptom severity scale* | 2.88 (0.65) | 0 |
| Functional status scale | 2.38 [1.75, 2.88] | 0 |
| VAS |  |  |
| Pain during loading | 55.50 [21.75, 74.00] | 1 |
| Pain at rest | 50.50 [21.50, 63.25] | 1 |
| Average pain during the previous week | 56.50 [31.50, 71.25] | 1 |
| Hand function* | 50.00 [34.00, 71.50] | 2 |
| Satisfaction with the hand | 32.00 [19.75, 49.50] | 1 |
| B-IPQ |  |  |
| Consequences | 7.00 [6.00, 8.00] | 1 |
| Timeline | 5.00 [3.00, 7.00] | 1 |
| Personal control | 4.00 [2.00, 6.00] | 1 |
| Treatment control | 9.00 [8.00, 10.00] | 1 |
| Identity | 7.00 [5.00, 8.00] | 1 |
| Concern | 5.00 [3.00, 7.00] | 1 |
| Understanding* | 8.00 [7.00, 10.00] | 1 |
| Emotional response | 5.00 [2.00, 6.00] | 1 |
| CEQ |  |  |
| Credibility score | 25.00 [23.00, 27.00] | 1 |
| Expectancy score* | 24.40 [21.80, 25.40] | 1 |
| PHQ-4 |  |  |
| Anxiety score | 0.00 [0.00, 2.00] | 1 |
| Depression score* | 0.00 [0.00, 1.00] | 1 |

* Variables used as input in the prediction model
