# Supplementary material for: Algorithm Versus Expert: Machine Learning Versus Surgeon-Predicted Symptom Improvement After Carpal Tunnel Release
Source: Neurosurgery. 2024 Feb 1;95(1):110–7. doi: 10.1227/neu.0000000000002848 (PMC11155572; doi:10.1227/neu.0000000000002848)
Supplement: SUPPLEMENTARY MATERIAL [file neu-95-110-s005.docx]

***Supplementary Table 3***. VAS scores and satisfaction scores six months after surgery of patients who reached the MCID and those who did not, to provide context on the relevance of the MCID. NB: Not all included patients completed all follow-up questionnaires, which resulted in some missing data in the VAS scores and satisfaction scores six months after surgery.

|  | Reached MCID | Did not reach MCID | P-value |
| --- | --- | --- | --- |
| VAS | N=54 | N=19 |  |
| Pain during loading | 6.00 [1.00, 19.50] | 32.00 [13.00, 53.00] | 0.006 |
| Pain at rest | 2.00 [0.00, 6.50] | 19.00 [7.00, 37.00] | 0.001 |
| Average pain during the previous week | 4.50 [0.00, 20.75] | 25.00 [11.50, 50.00] | 0.005 |
| Hand function | 90.00 [67.00, 99.00] | 80 [67.00, 94.00] | 0.26 |
| Satisfaction with the hand | 88.00 [69.00, 97.75] | 71.00 [50.50, 92.50] | 0.26 |
| Satisfaction with treatment results,  n (%) | N=54 | N=18 | <0.001 |
| Excellent | 26 (48) | 4 (22) |  |
| Good | 21 (39) | 2 (11) |  |
| Fair | 6 (11) | 8 (44) |  |
| Moderate | 0 (0) | 2 (11) |  |
| Poor | 1 (2) | 2 (11) |  |
